# Hypnosis and cognitive behavioral therapy with online sessions to reduce fatigue in patients undergoing chemotherapy for a metastatic colorectal cancer: Rational and study protocol for a feasibility study

**DOI:** 10.3389/fpsyg.2022.953711

**Published:** 2022-07-27

**Authors:** Louise Baussard, Florence Cousson-Gélie, Marta Jarlier, Elodie Charbonnier, Sarah Le Vigouroux, Lucile Montalescot, Chloé Janiszewski, Michele Fourchon, Louise Coutant, Estelle Guerdoux, Fabienne Portales

**Affiliations:** ^1^UNIV. NIMES, APSY-V, F-30021 Nîmes Cedex 1, Languedoc-Roussillon, France; ^2^Université Paul Valéry Montpellier 3, Laboratoire Epsylon EA4556, Languedoc-Roussillon, France; ^3^Institut du Cancer de Montpellier – Université de Montpellier, Languedoc-Roussillon, France; ^4^Institut Desbrest d'Epidémiologie et de Santé Publique, INSERM, Université de Montpellier, Languedoc-Roussillon, France

**Keywords:** fatigue, cancer, intervention, hypnosis, cognitive behavioral therapy, protocol, feasibility

## Abstract

**Background:**

In metastatic colorectal cancer (CRCm), fatigue is pervasive, reduces quality of life, and is negatively associated with survival. Its course is explained in part by psychosocial variables such as emotional distress, coping strategies, or perceived control. Thus, to reduce fatigue, psychosocial interventions appear to be relevant. In some cancers, Cognitive Behavioral Therapies (CBT) reduce fatigue. Hypnosis is also used as a complementary therapy to reduce the side effects of cancer. While CBT requires specific training often reserved for psychologists, hypnosis has the advantage of being increasingly practiced by caregivers and is therefore less expensive (Montgomery et al., 2007). On the other hand, CBT and hypnosis remain understudied in the CRC, do not focus on the symptom of fatigue and in Europe such programs have never been evaluated.

**Objectives:**

Implementing an intervention in a healthcare setting is complex (e.g., economic and practical aspects) and recruiting participants can be challenging. The primary objective will therefore be to study the feasibility of two standardized interventions (hypnosis and CBT) that aim to reduce fatigue in patients with CRCm treated in a French cancer center.

**Methods and design:**

A prospective, single-center, randomized interventional feasibility study, using mixed methods (both quantitative and qualitative). A total of 60 patients will be allocated to each intervention group [Hypnosis (*n* = 30) and CBT (*n* = 30)]. Participants will be randomized into two parallel groups (ratio 1:1). Both programs will consist of 6 weekly sessions focusing on the CRF management over a period of 6 weeks. Trained therapists will conduct the program combining 3 face-to-face sessions and 3 online sessions. The feasibility and experience of interventions will be evaluated by the outcome variables, including the adhesion rate, the reasons for acceptability, relevance or non-adherence, the satisfaction, the fatigue evolution (with ecological momentary assessments), and the quality of life. All questionnaires will be self-assessment using an online application from the cancer center.

**Discussion:**

Results will highlight the barriers/facilitators to the implementation of the program and the relevance of the program to the patients, and will be used to generate hypotheses for a randomized control trial.

**Clinical trial registration:**

ClinicalTrials.gov Identifier: NCT04999306; https://clinicaltrials.gov/ct2/show/NCT04999306.

## Introduction

In cancer patients, fatigue appears to be one of the most frequent and persistent symptoms (NCCN National Comprehensive Cancer Network, 2017). Cancer-related fatigue (CRF) has been defined as a distressing, persistent, subjective sense of physical, emotional, and/or cognitive tiredness or exhaustion related to cancer and/or cancer treatment that is not proportional to recent activity and interferes with usual functioning (NCCN National Comprehensive Cancer Network, 2017). Patients consider it severe and intense (Stone et al., 2000). Fatigue can have important psychosocial consequences such as reduced daily activities, and decreased quality of life (Lawrence et al., 2004; Forlenza et al., 2005).

Patients with metastatic colorectal cancer (CCRm) may follow several chemotherapy cycles. They follow months or even years of treatments, leading to many side effects (Wagland et al., 2015) and involving a decrease in Quality of Life (QoL) and functional status (Mayrbaeurl et al., 2016). In patients with colorectal cancer, fatigue ranks first among chemotherapy-related adverse events, with 75% of patients presenting a grade 3-4 with physical and psychological consequences (Mitry et al., 2010; Mota et al., 2012; Vardy et al., 2014). Then, metastatic progression and the increasing number of treatments are also aggravating factors (Peria et al., 2012), with a study reporting that 16% of patients in a clinical trial have fatigue graded at ¾, which corresponds to a very intense fatigue (Miyamoto et al., 2016).

**Figure 1:**
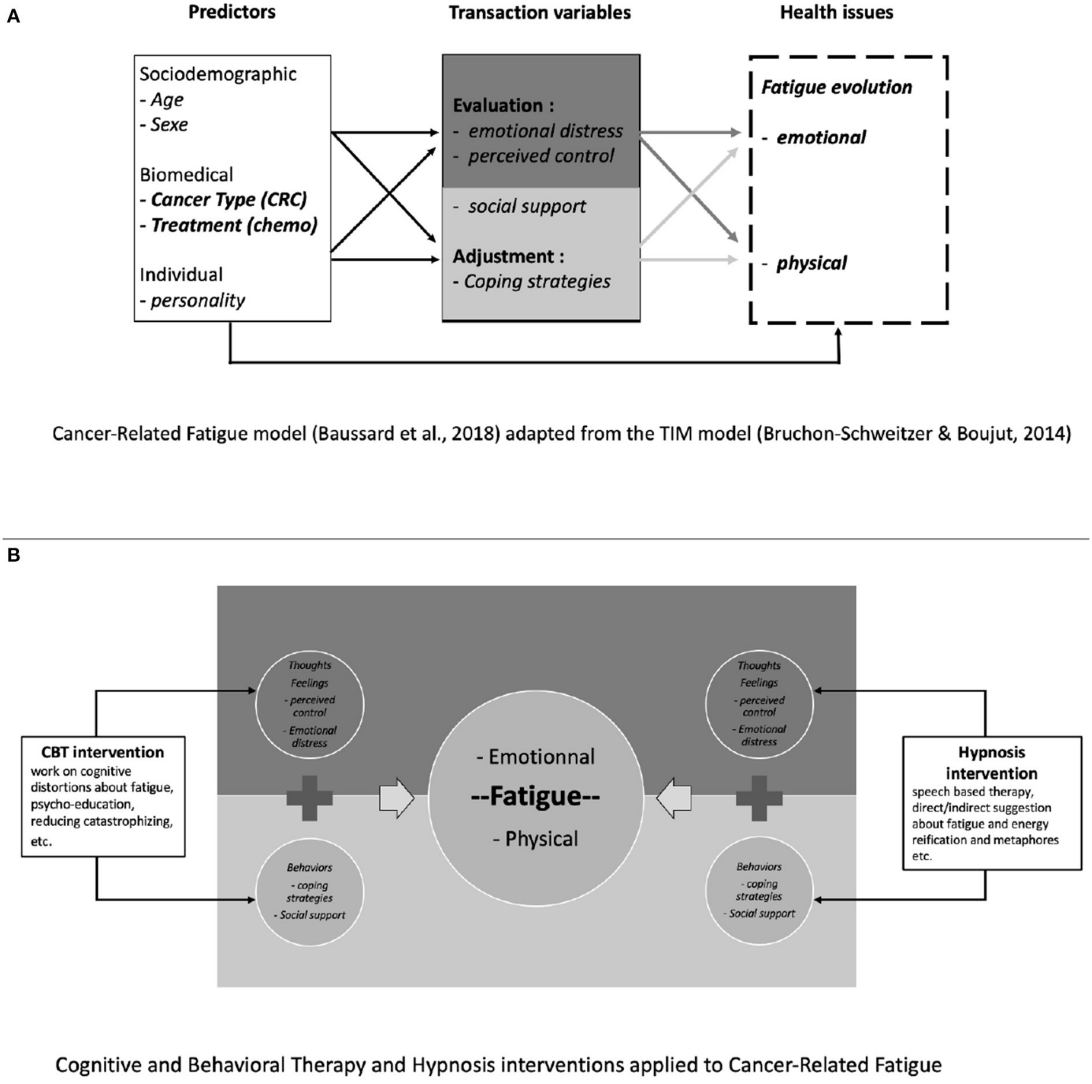
**(A,B)** From theory to practice–psychosocial interventions (CBT and hypnosis) applied to the determinants of cancer-related fatigue.

CRF is not only a consequence of the disease or the treatments. Indeed, some authors have made recommendations for psycho-oncology researchers to study the role of personality in developing or coping with CRC cancer and the associated QoL (Sales et al., 2014). To better understand the psychological factors associated with fatigue in CRCm patients, a recent study highlighted distinct trajectories of fatigue in this specific population during the course of chemotherapy (Baussard et al., 2022). That study was theoretically based on the Transactional, Integrative and Multifactorial (TIM) model (Bruchon-Schweitzer and Boujut, 2014) which suggests that health issues are explained by sociodemographic, medical and dispositional variables (such as personality) and by contextual variables (such as resources, e.g., coping strategies, perceived control, etc.). As shown in Figure 1A, Baussard (2018) have applied this model to the specific CRF and its evolution during the treatment. Results suggest that in CRCm patients undergoing chemotherapy, four distinct fatigue profiles were identified. The probability of belonging to each of these trajectories is explained by variables considered by the TIM model: tired patients are those reporting more emotional distress, little perceived control or inadequate coping strategies (Baussard et al., 2022). These results corroborate the literature underlying the relationship between emotional distress and fatigue (Blesch et al., 1991; Mols et al., 2012; Yennurajalingam et al., 2016), even in CRC patients (Mota et al., 2012; Tung et al., 2016). To our knowledge, it remains, however, the only study that have investigated psychological resource variables (such as coping, perceived control, etc.), suggesting that fatigue may be a consequence of a psychological maladjustment and imply that psychosocial interventions may be proposed to patients.

To address fatigue in cancer patients, the NCCN National Comprehensive Cancer Network (2017) highlights several types of interventions such as physical activity and psychosocial interventions. In 2008, Kangas et al. conducted a meta-analysis using data from 57 studies of non-medication therapies in cancer. The results indicated that the most effective strategies were physical activity and psychosocial interventions such as Cognitive and Behavioral Therapies (CBT), with no major difference in effectiveness between these two types of intervention (Kangas et al., 2008; Mustian et al., 2017). CRC patients reported barriers to physical activity programs, including age, pain, or respiratory and cardiovascular comorbidities (Fisher et al., 2016). Thus, we believe that psychosocial interventions have value and another meta-analysis in breast cancer patients reported that, like exercise programs, CBT, relaxation, counseling and hypnosis are beneficial in improving fatigue among patients with breast cancer (Duijts et al., 2011).

CBT for cancer patients includes therapeutic strategies focused on stress management (e.g., related to the announcement, surgery, treatment), problem solving to better cope with difficult situations, or cognitive restructuring (e.g., modification of dysfunctional cognitions associated with cancer) (Page et al., 2006; Berger et al., 2009; Chaloult et al., 2010; Cousson-Gélie et al., 2011). As shown in Figure 1B, CBT could be applied directly to CRF, by working on the thoughts and cognitive distortions associated with the symptom of fatigue. It aims to reduce emotional distress and encourage the development of a sense of control. Müller et al. (2021) found that increased self-efficacy and decreased fatigue catastrophizing, focusing on symptoms, perceived problems with activity and depressive symptoms mediate the reduction of fatigue brought by CBT. Finally, many studies have shown the effectiveness of CBT on fatigue in cancer patients (Page et al., 2006; Berger et al., 2009; Morin et al., 2009; Heckler et al., 2016) but never in patients undergoing chemotherapy for a metastatic CRCm.

In addition, the French Academy of Medicine suggest the interest of medical hypnosis in the management of the side effects of chemotherapy, such as nausea, vomiting or CRF (Bontoux et al., 2013). Hypnosis is defined as “a state of consciousness involving focused attention and reduced peripheral awareness characterized by an enhanced capacity for response to suggestion” (Elkins et al., 2015). Studies in breast cancer patients show a positive effect of hypnosis combined with CBT on symptoms of distress and physical fatigue (Cramer et al., 2015; Roe et al., 2016). A recent literature review found that only six studies investigated the effectiveness of hypnosis (not combined with another therapy) on cancer symptoms and CRF (Baussard et al., 2020). Results indicate that four studies reported effectiveness of hypnosis: two studies when the session is conducted by a therapist (Montgomery et al., 2007; Jensen et al., 2012), with effect sizes (*d*) equal to 0.80 and 2.05 respectively, and two studies reported significant efficacy of auto-hypnosis (listening audio-tape) (Bragard et al., 2017; Gregoire et al., 2017). However, these four studies remain in the minority and have limitations in generalizing the results. First, the samples are too small with *n* = 8 in the study by Jensen et al. (2012) and *n* = 68 for the studies by Bragard et al. (2017) and Gregoire et al. (2017). Second, all of these studies focused on women with breast cancer, which is a specific population. The study of Montgomery et al. (2007), a two-arm RCT with *n* = 100 for the hypnosis intervention seems more robust but assessed post-surgery fatigue, which again is very specific. Thus, this review of the international literature concludes that there is a lack of studies on the effectiveness of hypnosis for CRF (Baussard et al., 2020).

As shown in Figure 1B, as well as CBT, hypnosis could be used to manage CRF, by suggesting ways to regain energy or rest, or by working on a “safe place” to enhance relaxation, etc. The aim is to modify the patient's representations and to help her/him to tame this symptom. In addition, hypnosis has the advantage of being increasingly practiced among professional caregivers in hospitals (Montgomery et al., 2007; Gueguen et al., 2015), and also accepted by the patients (Bragard et al., 2017) which facilitates its implementation.

Thus, the relative efficacy of hypnosis and CBT on CRF has been described in oncology, but very few of these interventions have been conducted specifically on CRCm patients. The difficulties in implementing such programs in the care pathway underlines the importance of conducting this type of study. To our knowledge, there is no study proposing CBT or hypnosis in patients with CRC to reduce CRF. Since the implementation of two psychosocial intervention programs in a health care center is complex, and there is a need to develop standardized interventions for a specific population (CRCm patients), it was decided to first conduct a feasibility study. van Lankveld et al. (2018) suggest that feasibility studies should assess interest and willingness of patients to receive professional psychosocial care. To go further, evidence-based practice, defined as “the conscientious, explicit and judicious use of current best evidence in making decisions about the care of individual patients” (Trinder, 2008) has become an important feature of health care systems and health care policy.

Furthermore, several studies and meta-analyses underline a better effectiveness of face-to-face sessions (Montgomery et al., 2002; Schnur et al., 2008; Askay et al., 2009), and individual sessions rather than group sessions (Cousson-Gélie et al., 2011). We therefore propose individual face-to-face sessions. Beside, the programs in this study are intended to respect the chemotherapy pathway of CRC patients (i.e., a treatment every 2 weeks). This approach seems to be essential to allow a good implementation within the hospital, and not to induce an additional fatigue in patients by imposing them an additional travel.

This will also allow us to gather precise indicators (barriers/facilitators) regarding the implementation of psychosocial interventions in cancer centers, in order to meet the expectations of an increasingly personalized medicine, insisting on the essential place of support care.

### Objectives

The main objective will be to assess the feasibility of two standardized interventions for fatigue reduction in patients with CCRm. One of the two interventions will propose a dedicated CBT and the other an hypnosis intervention. It seems essential to assess the proportion of volunteers to participate in these interventions as well as the acceptability of the programs, the method of data collection and the barriers/facilitators to the implementation of these two interventions. Therefore, several secondary objectives are planned:

1. To assess acceptance to participate in the study and reasons for refusal;
2. To identify the reasons for non-adherence to the program;
3. To highlight the barriers/facilitators to the implementation of the protocol;
4. To evaluate the relevance of the program (adapt to the patients' needs);
5. To assess fatigue (pre- and post-intervention) and its evolution;
6. To describe the QoL at baseline and after the intervention.

Although this is a feasibility study, patients participating in either program will benefit from a possible improvement in their fatigue status. There are no risks to report.

## Method and analysis

### Design

This is a prospective, single-center, randomized interventional feasibility study. This feasibility study proposes the implementation of two intervention programs (CBT and Hypnosis) to reduce CRF. Thus, patients included in the study will be randomized into two parallel groups (ratio 1:1). Procedure is detailed below. This research will use mixed methods (both quantitative and qualitative analyses) and data triangulation. Indeed, to increase the ecological validity of the study it is necessary to cross different types of data, analyses and/or participants (Salkind, 2010). In this study, combining a quantitative approach to program satisfaction with a qualitative approach to program experience allows us to answer complex research questions while taking advantage of the benefits and minimizing the limitations of qualitative or quantitative studies alone.

### Population

#### Recruitment

Patients will be included in a French regional anticancer center. We plan to include 60 patients who will be allocated to each intervention group [i.e., Hypnosis (*n* = 30) and CBT (*n* = 30)]. The sample size will not be based on a power analysis but on the estimated recruitment capacity in the cancer institute.^1^ A recruitment of 30 patients per intervention group would allow us to estimate in each group a proportion of at least 80% of patients adhering to each intervention, with their 95% confidence intervals, of amplitude 0.28 (Machin et al., 2009).

#### Eligibility

Inclusion criteria will be as follows:

1) Age ≥ 18 years
2) WHO performance status ≤ 2
3) Progressive colorectal adenocarcinoma after first line metastatic chemotherapy
4) Able to understand and read French
5) Visual Analog Scale for fatigue ≥ 4: based on our previous work on metastatic CRC patients where 48% complained of moderate fatigue throughout treatment, and since our variable of interest is fatigue, we decided to include patients already fatigued (4/10 on VAS).
6) Patients starting a second or third line of metastatic chemotherapy
7) Patients who signed the informed consent
8) Patients subscribed to a French Social Security system

The criteria for non-inclusion will be as follows:

1) Patients who do not have a telephone or devices that allow remote monitoring of sessions at home
2) Presence of brain metastases
3) Chronic pain evolving for more than 3 months and on morphine
4) Patients who used to and/or have a regular and habitual practice in meditation, or in relaxation techniques such as yoga, hypnosis or sophrology
5) Medical (neurological, psychiatric, etc.) or psychological conditions that do not allow participation in the protocol (filling out the questionnaires and the booklet, as well as following the sessions)
6) Deaf patient without hearing aids
7) Patient under guardianship or legal protection

#### Description of the programs

Both programs consist of 6 weekly sessions, each lasting ~1 h (shorter in the hypnosis arm), of CRF management over a period of 6 weeks. They focus on CRF and its psychosocial determinants (see **Table 2**):

- Session 1: Patient Education about CRF
- Session 2: Work on perceived control
- Session 3: Work on emotional regulation
- Session 4: Work on social support during illness
- Session 5: Working on coping strategies
- Session 6: Review of previous exercises/sessions, synthesis

#### CBT

In order to standardize the program, the content of the CBT intervention was inspired by previous studies (Gielissen et al., 2006, 2007; Poort et al., 2017) and based on previous results focusing the psychosocial determinants of fatigue in metastatic CRCm patients (Baussard et al., 2022). In Gielissen et al. (2006) study, the rationale of the intervention was based on the model of precipitating and perpetuating factors, where each session focused on fatigue perpetuating factors such as coping with the experience of cancer, fear of disease recurrence, dysfunctional cognitions concerning fatigue or negative social interactions. Inspired by these studies, each session of the CBT program will focus on fatigue determinants (Table 2).

#### Hypnosis

Hypnosis, as a speech-based therapy, is difficult to standardize. One way to standardize the protocol is to distribute audio-scripts to patients to listen to at home independently (Bragard et al., 2017; Gregoire et al., 2017). However, this way has two main limitations. First, each patient has her/his own vocabulary, that makes uncertain the standardized and general speech-based efficacy. Then, the benefit from the session as if a therapist were present, compared with the benefit from a session with a therapist, may differs (as there is no way to ensure the state of hypnosis or the duration of the session, for example). The (Jensen and Patterson, 2006; Jensen et al., 2008, 2012) ‘s hypnosis included sessions based on a written script that each clinician read to the patients, although minor wording changes were allowed to facilitate verbal flow. In 2013, Montgomery et al. (2013) gave advices for setting up a standardized hypnosis program to facilitate its evaluation: introducing of the session; inducting; working on metaphors or making specific suggestions and then instructing for self-hypnosis or anchoring. Moreover, Grégoire et al. (2022) published a study protocol where hypnosis was proposed in a face-to-face and in a standardized way. Taken as a whole, these works encourage the creation of a specific program with exercises specifically designed to work on a problem-related fatigue as it is the case in the CBT intervention. Description of hypnosis sessions and associated fatigue variables are presented in Table 1.

1. The proportion of patients giving consent to participate in the study compared with the number of patients approached for the study, and the reasons for refusal to participate;
2. The qualitative reasons reported by the patients for non-adherence to the program (for patients dropping out of the program);
3. The qualitative barriers/facilitators reported by the patients to the program implementation;
4. (a) The score of the Consumer Satisfaction Questionnaire (CSQ-8) – (Kapp et al., 2014). This scale allows the quality-of-care assessment and will evaluate the acceptance and relevance of the programs.
(b) The score on the Session Evaluation Questionnaire (SEQ in order to assess the weekly satisfaction about the sessions. It takes 5 minutes and consists of 22 items. Each item represents two bipolar adjectives on which the patient ranks at the end of a therapeutic consultation. For the purposes of this study, we will consider only the first factor, the 11 items on the feeling of the session (pleasant interview, powerful, superficial, etc.) (Stiles, 1980).
5. (a) The scores on the Multidimensional Fatigue Inventory (MFI-10) - (Baussard et al., 2018). This 10-items short scale is validated in cancer patients and assess physical, emotional and cognitive fatigue.
(b) The scores on the VAS fatigue (Baussard et al., 2017). This VAS is adapted on the phone application (≪ Mon Essai Patient ≫) for the study needs, while respecting its original form. Patients are asked to indicate, with a cursor going from 0 to 10, their state of fatigue, whether physical (I lack energy) or psychological (I feel weariness).
6. The scores on the Quality of Life (QLQ-C30 – EORTC) - (Aaronson et al., 1993). The QLQ-C30 incorporates nine multi-item scales: five functional scales (physical, role, cognitive, emotional, and social); three symptom scales (fatigue, pain, and nausea and vomiting); and a global health and quality-of-life scale. The EORTC QLQ-C30 was extensively validated in European languages, including French;

**Table 1:** Description^1^ of the sessions for both programs.

	**Hypnosis**	**CBT**
Session's themes		
Each session ends with a debriefing and Questions/Answers		
Fatigue education	Program presentation Fatigue education Short hypnosis session: “Safe place” induction Questions/Answers	Program presentation Fatigue education Introduction to CBT Questions/Answers
Perceived control	Induction with sensory data collection (fatigue as a deep feeling of tiredness) Miracle question and search for exception based on Solution-focused therapy	Discussion about patient's representations of fatigue and cancer Exercise on causal attributions (internal, external and hazard) To think about action to implement to regain control over fatigue
Emotional regulation	Induction on breathing Safe place and work on emotion observations and rating Anchoring the feeling of wellbeing	To identify feelings and associated thoughts To list problematic situations To notice dysfunctional thoughts and searching for alternative thoughts
Social support	Questioning about relatives support and induction on today fatigue sensation Rossi's mirroring hands to identify people who are social resources	Presentation of social support and its dimensions To identify people resources for each type of support To learn how communicate needs and assertiveness
Coping strategies	Induction with body scan to focus on a specific site where fatigue is intense (e.g., shoulders or legs) Work on metaphors and reifying the symptom	Presentation of coping strategies To think about patient's behaviors and coping efficacy (Cunji's circle) To consider alternative behaviors
Synthesis	Feedback on the program and on the exercises that need to be repeated To see with the patient where he/she stands	Feedback on the program and on the exercises that need to be repeated To see with the patient where he/she stands

*For the content of the programs (CBT and Hypnosis), each therapist will follow a guide where each session is described (how to introduce the session, exercises during the session, at-home practices, etc.). These built guides are the property of the authors and can be provided on an argued request of interested parties (replications, systematic review)*.

*Each session from 2 to 6. Begins with a debriefing on the previous session (feedback on home exercises for CBT; feedback on experience if self-hypnosis for Hypnosis)*.

### Measures

In this feasibility study, the primary endpoint is the proportion of patients who have adhered to the proposed intervention program (both CBT and hypnosis programs include 6 sessions). A patient will be considered adherent if she/he participated in at least 4 of the 6 proposed intervention sessions. Adherence is defined in the same way in each intervention group. The choice to consider adherence to the programs at 4 out of 6 sessions is a commonly accepted practice in non-medication intervention studies (Brebach et al., 2016). Thus, patients participating in the study will have to receive at least 4 sessions (addressing key issues related to fatigue) in order to consider the program beneficial (hypnosis or CBT).

There are secondary quantitative and qualitative endpoints:

#### Semi-directed interviews

Qualitative interviews are planned to assess the relevance of the programs. The interviews will be semi-structured to obtain as much information as possible. A first general question will ask about their general opinion of the program. Then, they will be asked to talk about their reasons for agreeing to participate in the program, as well as information about the implementation. Patients will answer whether the program seemed suitable or not and finally, whether they would recommend it to others. Each question is as broad as possible, and then participants will be prompted if more detail is needed. Patients who would like to end their participation in the program prematurely will also be invited to participate in these interviews, as the reasons for non-adherence are among the evaluation criteria. It is difficult to estimate the time, some patients will speak little, others elaborate more. For open-ended questions, we estimate a minimum of 20 min and a maximum of 1 h.

#### Repeated measures

Our protocol involves an ecological momentary assessment (EMA) of fatigue, ideally at the same time of day. This daily measurement of fatigue is planned from T0 to T1, i.e., 7 weeks (49 assessments). Daily fatigue assessment takes 2 min with the VAS, one for each item (Baussard et al., 2017).

Weekly satisfaction about the sessions takes 5 min and will be assessed by The Session Evaluation Questionnaire (SEQ). Each 22 item represents two bipolar adjectives on which the patient ranks at the end of a therapeutic consultation. For the purposes of this study, we will consider only the first factor: 11 items on the feeling of the session (pleasant interview, powerful, superficial, etc.) (Stiles, 1980).

### Procedure

Three time points will be considered: T0 corresponds to the patient' inclusion in the study (patient agreement and completion of the study questionnaires), T1 corresponds to the end of the intervention after 6-weeks of intervention (semi-directive interviews on the acceptability of the program and completion of the questionnaires), and T2 corresponds to a follow-up visit 3 months after the intervention (questionnaires).

During the medical consultation with the oncologist, eligible patients will be identified. After oncologist approval, only inclusion/non-inclusion criteria (including fatigue VAS) will be retrieved by the investigator. If eligible, a patient will receive the information letter and consent, and will be asked to return it for cure of chemotherapy N°1 (T0).

The investigator will collect the sociodemographic (including the Score EPICES) and the medical variables. She/he will also present a web application (smartphone or tablet), available on AppStore or PlayStore (“*Mon Essai Patient*”) from the company Exolis. Patients who are not comfortable with these tools have the option of having a paper format. This application allows to collect every data from the questionnaires, encourages the daily or weekly assessment and limits missing data because patients will receive a notification when a questionnaire must be completed. Then a clinical research associate will give the different self-questionnaires to the patient, responding her/his questions if necessary. As shown Table 2, the patient will fill out, during ~30 min, the MFI-20, theQLQ-C30, and the VAS fatigue.

**Table 2:** Measurements used in the study.

**Measures**	**Screening**	**T0**	**6 weeks interventions**	**T1**	**T2**
Inclusion/non-inclusion criteria	X				
Information letter and consent		X^a^			
Sociodemographics and medical variables		X		X^b^	
EPICES score		X^c^			
Fatigue and Quality of Life:					
6. VAS fatigue (physical/emotional)	X	X	X^c^ Daily assessment		X^c^
7. MFI-20		X^c^		X^c^	X^c^
8. QLQ-C30		X^c^		X^c^	X^c^
Feasibility					
9. SEQ			X^c^		
			Weekly assessment		
10. ISQ-8				X^c^	
11. Qualitative interviews				X^c^	

a *Information letter and consent are delivered at screening and retrieved if inclusion at T0*;

b * if progression, relapse or change of treatment*;

c * E-health application; MFI-20, Multidimensional Fatigue Inventory; QLQ-C30, Quality of Life Questionnaire – Cancer; Score EPICES, precariousness and social inequalities in health; SEQ, session evaluation questionnaire; CSQ-8, satisfaction questionnaire about interventions*.

Randomization procedure. If the patient meets all the inclusion criteria and return the consent at T0, Biometrics Unit will proceed with the randomization and return the assigned treatment arm to the investigator. This form should also be completed and submitted for patients who meet the inclusion criteria but do not agree to participate in the study. A specific section of the form will be dedicated to them in order to register these patients (this will allow to answer the first secondary objective; i.e., the acceptance of participation in the study). The randomization procedure using the random block method will allocate the Hypnosis or CBT treatments with a 1:1 ratio and will be stratified according to two factors with two modalities: the number of lines of chemotherapy (2nd or 3rd line) and the management by the support care platform in the hospital (yes, no). Regarding stratification, the number of lines of treatment may have an impact on the primary endpoint (increase in fatigue) since it implies a non-response to treatment, a progression of the disease, and possibly a deterioration in the patient's condition. Secondly, the patients received at the Center may be offered support care as part of their standard care (*e.g.*, nutritional follow-up), and this may also have an impact on the primary endpoint (*e.g.*, reduction in fatigue). Therefore, these two factors will be considered as a stratification factor.

**Figure 2:**
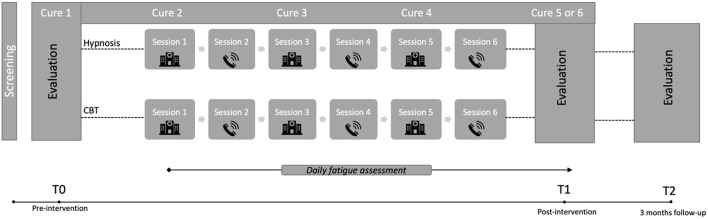
Study design with two-arms randomization (hypnosis and CBT), 6 sessions each. Flexibility in terms of sessions is foreseen, i.e., a patient may have a session shifted by 1 or 2 weeks depending on its WHO performance status and/or the therapist's availability. This takes into account the toxicities, need to always have the same practitioner for the 6 sessions, and the reality of the field (absences, vacations, etc.).

As shown in flow-chart (Figure 2), an included patient will be assigned to one of two intervention programs. Either hypnosis with 6 standardized sessions (1/week) of ~30–45 min or CBT with 6 standardized sessions (1/week) of about 1 h. In order to respect the chemotherapy pathway of CRCm patients, one session will be done face-to-face on the day of the treatment, and the following session will be offered remotely by telephone or videoconference (at a distance) when the patient is at home during the week without chemotherapy. For the three remote sessions of the intervention, patients can choose between telephone or videoconference therapy. If patients choose video, the sessions will be conducted using a secure tele-health platform called *Starleaf* (https://starleaf.com/about-us/). This company was Founded in 2008 in Cambridge and provides secure meetings, messaging and calling to enterprises worldwide. Thus, the first session of a program will take place face to face, during treatment cure N°2 (i.e. after a respected delay after the proposal to participate in the protocol), the third and the fifth respectively during chemotherapy N°3 and N°4 in hospital. The second, fourth and sixth sessions will take place remotely. Three therapists (one for the hypnosis and two for the CBT program) will implement the interventions and all patients will receive an individual notebook with explanations of the program, fatigue education, and blank pages to take notes after each session. The mail of the clinical research assistant will also be presented here if any questions arise between two sessions.

At T1, after the interventions, patients will be asked to complete again the QLQ-C30, MFI, EVA fatigue and the ISQ-8 (see Table 2). They also will be interviewed by a trained psychologist about their acceptation of the program with semi-directed interviews. The interviews will be semi-structured to obtain as much information as possible. A first general question will ask about their general opinion of the program. Then, they will be asked to talk about their reasons for agreeing to participate in the program, as well as information about the implementation. Patients will answer whether the program seemed suitable or not and finally, whether they would recommend it to others. Each question will be as broad as possible, and then participants will be prompted if more detail is needed. Patients who would like to end their participation in the program prematurely will also be invited to participate in these interviews, as the reasons for non-adherence are among the evaluation criteria.

At the end, regardless of the inclusion arm, patients will receive one intervention over a period of 6 weeks and will be re-interviewed 3 months after the procedure (T2) on their level of fatigue and quality of life, representing 5 months involvement in total.

### Statistical considerations

The primary outcome will be analyzed in each intervention group. The proportions of patients who adhered to each intervention program (Hypnosis and CBT) will be presented with their two-sided 95% confidence intervals (95% CI), as the proportion of patients giving consent to participate in the study among the eligible patients at the screening who were offered the study. All statistical analyses of the secondary criteria will be done by group. Categorical variables will be described by the number of observations (N) and the frequency (%). Missing categories will be counted. Quantitative variables will be described by the number of observations (N), mean, standard deviation, median, minimum, and maximum. In case of missing data no imputation method will be used.

For the comparison of qualitative variables, a chi-square test or a Fisher's exact test will be used if the theoretical number of observations is <5. For the comparison of quantitative variables, a Student's *t-*test or a Kruskal-Wallis test will be used. For comparisons between two time points a Wilcoxon test for paired samples will be used. The correlation between the VAS values for fatigue and the scores on the MFI-10 questionnaire will be studied using Pearson's correlation coefficient or Spearman's correlation coefficient. The evolution of VAS fatigue values over the course of treatment will be modeled using linear mixed models to account for the repeated nature of the measurements. The analysis of the QLQC-38 questionnaire will be performed according to the EORTC guidelines.

All statistical tests will be two-sided and the significance level is set at 5% (i.e., *p* < 0.05). Statistical analyses will be performed with STATA v16 software and a statistical report will be provided according to the current model.

Program satisfaction will be assessed on the basis of the CSQ-8 score, SEQ evaluation of each session and put into perspective with the individual semi-structured interviews analyzed with QDA Miner. This is a mixed methods and qualitative data analysis software developed by Provalis Research. The program was designed to assist researchers in managing, coding and analyzing qualitative data. QDA Miner was first released in 2004 and the latest version 6 was released in September, 2020.

## Discussion

We conduct this study with a view to evidence-based practice. It is an approach that limits clinical uncertainty, based on a tripartite evaluation taking into account the patient's point of view, the clinical experience and scientific evidence (Rycroft-Malone et al., 2004). Evidence-based practice in psychology (EBPP) promotes effective psychological practice by, among other things, applying empirically supported interventions (APA Presidential Task Force on Evidence-Based Practice, 2006).

In order to better understand the needs of patients and to know the possibilities of implementing such programs in a cancer center we decided to conduct a feasibility study. As we know, there is no such studies for managing fatigue in CRCm patients, except for Teo et al. (2020) who published a feasibility study on the implementation of CBT in patients with advanced CRC. Although the focus was not on fatigue, it does provide some insight into the acceptability of a psychosocial intervention in this population. They successfully recruited the intended sample (mean age 61 years; 62% men). Most patients (88%) completed all sessions and participants reported high rates of satisfaction (97%) and helpfulness (96%) of the intervention, which remains encouraging for our study. Patients use Complementary and Alternative Medicine (CAM) more often to increase the body's ability to fight the cancer or to improve physical wellbeing (Molassiotis et al., 2005; Lawsin et al., 2007; Wong et al., 2021). Studies investigating CAM show that CCR patients are increasingly turning to this type of medicine, but hypnosis is still not well-known (Molassiotis et al., 2005; Lawsin et al., 2007; Wong et al., 2021). It therefore seems interesting to understand the acceptability of CBT or hypnosis.

Several authors agree that longitudinal studies and Randomized-Controlled Trials should be set up to overcome limitations about methodology and standardization (Sood et al., 2007; Barsevick et al., 2013; Carlson et al., 2017). This pilot study, with two standardized interventions (including a hypnosis program) will encourage us to conduct a 3-arms RCT (CBT, hypnosis and control) to evaluate the efficacy of such therapies, and to answer the question of which therapy will benefit to the most vulnerable patients.

Many arguments allow to provide hypothesis about the results. First, there are distinct profiles of CRF in CRCm patients undergoing chemotherapy (Baussard et al., 2022): intense fatigue (6.51%), moderate fatigue (48.52%), no fatigue (33%), and increasing fatigue (11.83%). Secondly, CBT are costly in terms of time and investment, require specific training (psychologist) and seems difficult to implement in the care pathway or to offer them to all patients. Thirdly, hypnosis has the advantage of being more easily implemented in the treatment process because the nursing staff (nurses, caregivers, etc.) can be trained in it. Therefore, we would expect that those with severe fatigue (6%) would require more comprehensive treatment (CBT), whereas those with moderate fatigue (48%) would benefit from hypnosis alone. We aim to understand what is easiest to implement and most relevant to patients.

There are some others inherent limitations to the study that may negatively influence the rate of adherence to the program. The emotional burden of patients diagnosed with CRCm is very high, and the patients will have lot of information to integrate. In addition, the use of the e-health application is subject to technical hazards, in addition to the weariness it can create. While access to e-health is increasing worldwide, a gap remains between those who use between those who use these digital tools and those who do not use them (Wynn et al., 2020). Finally, an assessment of the costs for implementation of the programs on a routine basis should be planned.

Implementing these two programs will allow us to understand which management is the most effective and for which profiles of fatigued patients. This study could then inform us about the number of patients who need full and more expensive treatment (CBT), and ultimately allow institutions to respond to the demands of an increasingly personalized care and underlines the importance of evaluating the relevance of supportive care.

## Ethics statement

The studies involving human participants/the COLOFIGHT protocol were reviewed and approved by the Ile de France II French Ethical Committee (January 2022) and registered on ID-RCB n° 2021-A01031-40. The patients/participants will provide their written informed consent to participate in the study.

## Author contributions

LB and FC-G conceptualized the initial version of the project. LB, EC, SL, and LM developed the project methodology and built the CBT program. LB, MF, and LM constructed the hypnosis program. CJ and MJ helped with the implementation in the clinical research department. MJ with the statistical considerations of the project. EG and LC will conduct the CBT sessions, and MF the hypnosis sessions. All authors contributed to the design of the study presented here.

## Funding

This research received funding from the French National Cancer Institute (Grant INCA_16319) and was supported by the SIRIC Montpellier Cancer (Grant INCa-DGOS-Inserm_12553), which was not involved in study design; in the collection, analysis and interpretation of data; in the writing of the report; and in the decision to submit the article for publication.

## Conflict of interest

The authors declare that the research was conducted in the absence of any commercial or financial relationships that could be construed as a potential conflict of interest.

## Publisher's note

All claims expressed in this article are solely those of the authors and do not necessarily represent those of their affiliated organizations, or those of the publisher, the editors and the reviewers. Any product that may be evaluated in this article, or claim that may be made by its manufacturer, is not guaranteed or endorsed by the publisher.

## Footnotes

^1^The recruitment capacity and duration of the inclusions (12–18 months) are not only based on the recruitment capacity of the oncology department, but also take into account a practical aspect: the follow-up, which is the psychotherapists' capacity. Indeed, only 2 psychologists at the Center are trained in CBT and agreed to participate in the study, indicating that they will be able to follow up 2 patients per week each (i.e., 4 patients included every 6 weeks).

## Abbreviations

CAM: Complementary and Alternative Medicines
CRCm: colorectal cancer metastatic
CBT: Cognitive and Behavioral Therapy
CRF: Cancer-Related Fatigue
CSQ: Consumer Satisfaction Questionnaire
EBPP: Evidence-Based Practice in Psychology
INCa: French National Cancer Institute
MFI: Multidimensional Fatigue Inventory
NCCN: National and Comprehensive Cancer Network
QLQC: Quality of Life Questionnaire in Cancer
QoL: Quality of Life
SEQ: Session Evaluation Questionnaire
VAS: Visual Analog Scale
WHO: World Health Organization.
